# 4-({4-[1-(Methoxy­imino)eth­yl]anilino}(phen­yl)methyl­ene)-3-methyl-2-phenyl-1*H*-pyrazol-5(4*H*)-one

**DOI:** 10.1107/S1600536809048326

**Published:** 2009-11-21

**Authors:** Jian Yao, Su-Xia Gao, Wen-Kui Dong, Jun-Feng Tong, Shang-Sheng Gong

**Affiliations:** a School of Chemical and Biological Engineering, Lanzhou Jiaotong University, Lanzhou 730070, People’s Republic of China; b School of Environmental Science and Municipal Engineering, Lanzhou Jiaotong University, Lanzhou 730070, People’s Republic of China

## Abstract

In the title compound, C_26_H_24_N_4_O_2_, the dihedral angles between the central pyrazole ring and the other three benzene rings are 40.02 (3), 77.51 (5) and 55.72 (3)°. A strong intra­molecular N—H⋯O hydrogen bond forms a six-membered ring with an *S*(6) motif. In the crystal structure, a weak inter­molecular C—H⋯N inter­action with graph-set motif *R*_2_^2^(8) and C—H⋯O hydrogen bonds link each mol­ecule to three others, forming an infinite two-dimensional supra­molecular structure.

## Related literature

For background to 1-phenyl-3-methyl-4-benzoyl-5-pyrazolone, see: Lahiri *et al.* (2003 ▶). For related structures, see: Bomfim *et al.* (2005 ▶); Wang *et al.* (2008 ▶). For bond-length data, see: Allen *et al.* (1987 ▶). For graph-set notation, see: Bernstein *et al.* (1995 ▶). For the synthesis, see: Rafiq *et al.* (2008 ▶); Zhao *et al.* (2009 ▶); Dong *et al.* (2008 ▶). 
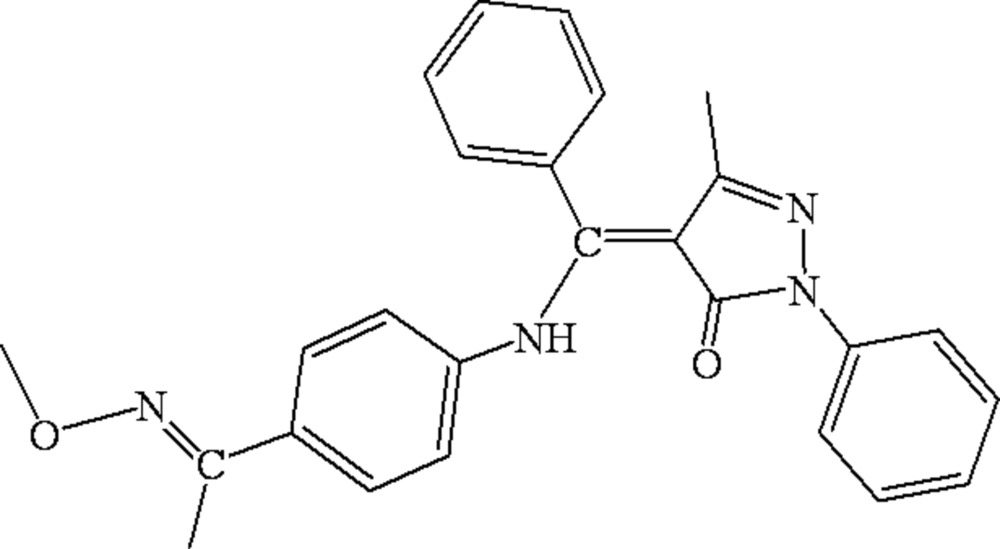


## Experimental

### 

#### Crystal data

C_26_H_24_N_4_O_2_
*M* *_r_* = 424.49
Triclinic, 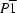

*a* = 7.3550 (6) Å
*b* = 11.1609 (13) Å
*c* = 14.9700 (12) Å
α = 68.536 (1)°
β = 76.654 (2)°
γ = 76.182 (2)°
*V* = 1096.46 (18) Å^3^
*Z* = 2
Mo *K*α radiation
μ = 0.08 mm^−1^
*T* = 298 K
0.50 × 0.49 × 0.10 mm

#### Data collection

Siemens SMART 1000 CCD area-detector diffractometer
Absorption correction: multi-scan (*SADABS*; Sheldrick, 1996 ▶) *T*_min_ = 0.960, *T*_max_ = 0.992
5654 measured reflections
3781 independent reflections
1861 reflections with *I* > 2σ(*I*)
*R*_int_ = 0.037

#### Refinement

*R*[*F* ^2^ > 2σ(*F* ^2^)] = 0.077
*wR*(*F* ^2^) = 0.246
*S* = 1.03
3781 reflections
290 parameters
H-atom parameters constrained
Δρ_max_ = 0.24 e Å^−3^
Δρ_min_ = −0.26 e Å^−3^

### 

Data collection: *SMART* (Siemens, 1996 ▶); cell refinement: *SAINT* (Siemens, 1996 ▶); data reduction: *SAINT*; program(s) used to solve structure: *SHELXS97* (Sheldrick, 2008 ▶); program(s) used to refine structure: *SHELXL97* (Sheldrick, 2008 ▶); molecular graphics: *SHELXTL* (Sheldrick, 2008 ▶); software used to prepare material for publication: *SHELXTL*.

## Supplementary Material

Crystal structure: contains datablocks global, I. DOI: 10.1107/S1600536809048326/fl2280sup1.cif

Structure factors: contains datablocks I. DOI: 10.1107/S1600536809048326/fl2280Isup2.hkl

Additional supplementary materials:  crystallographic information; 3D view; checkCIF report

## Figures and Tables

**Table 1:** Hydrogen-bond geometry (Å, °)

*D*—H⋯*A*	*D*—H	H⋯*A*	*D*⋯*A*	*D*—H⋯*A*
N3—H3⋯O1	0.86	1.95	2.683 (4)	142
C26—H26*C*⋯N4^i^	0.96	2.72	3.643 (9)	163
C9—H9⋯O1^ii^	0.93	2.67	3.398 (6)	135

Symmetry codes: (i) 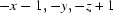; (ii) 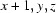.

**Fig. 1.**
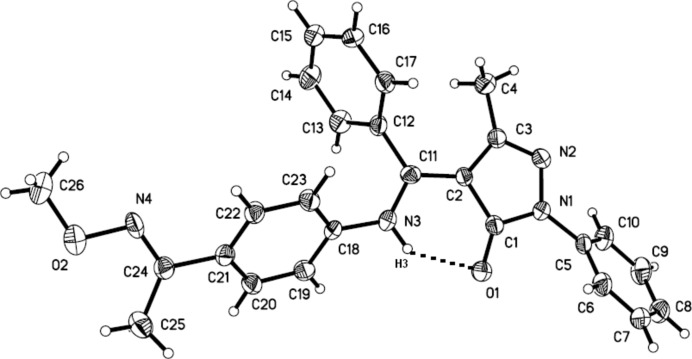
The molecular structure of the title compound with atom numbering scheme. Displacement ellipsoids for non-hydrogen atoms are drawn at the 30% probability level. Intramolecular hydrogen bond is shown as a dashed line.

**Fig. 2.**
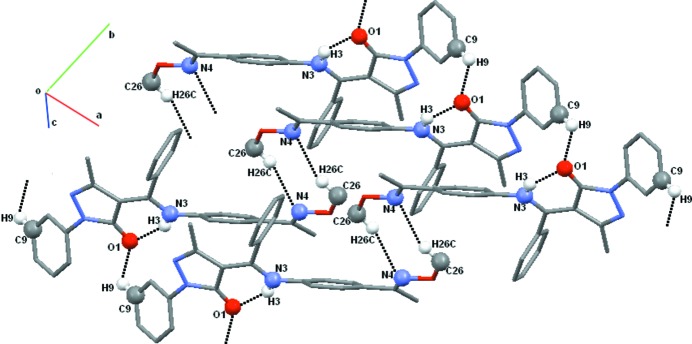
Part of two-dimensional supramolecular structure showing intermolecular hydrogen bonds interactions (dashed lines).

## supplementary crystallographic information

### Comment

1-Phenyl-3-methyl-4-benzoyl-5-pyrazolone (PMBP) is an efficient extractant of metal ions, and has the potential to form different types of compounds (Lahiri *et al.*, 2003). Some structures of oxime compounds, similar to the title compound (Fig. 1), formed by Schiff base reactions have been reported (Bomfim *et al.*, 2005; Wang *et al.*, 2008).

The title compound is a potential tridentate mono-oxime ligand. The bond lengths and angles in the molecule are within normal ranges (Allen *et al.*, 1987). In the molecule, the dihedral angles between the central pyrazole ring and the other three benzene rings (C5—C10), (C12—C17) and (C18—C23) are 40.02 (3)°, 77.51 (5)° and 55.72 (3)°, respectively.

In the crystal, a strong intramolecular N3—H3···O1 hydrogen bond forms a six-membered ring, producing a S(6) ring motif (Table 1, Fig. 1). A weak intermolecular C26—H26C···N4 interacation with the graph-set motif of *R*_2_^2^(8) (Bernstein *et al.*, 1995) and C9—H9···O1 hydrogen bonds, link each molecule to three others, forming an infinite two-dimensional supramolecular structure along the diagonal of the unit cell (Table 1, Fig. 2).

### Experimental

1-(4-Aminophenyl)ethanone *O*-methyl oxime (I) was prepared using 1-(4-aminophenyl)ethanone and methoxyamine (Rafiq *et al.*, 2008; Zhao *et al.*, 2009). The title compound was prepared according to a previously reported procedure (Dong *et al.*, 2008). To an ethanol solution (7 ml) of (I) (164.1 mg, 1.00 mmol) was added dropwise an ethanol solution (7 ml) of 1-phenyl-3-methyl-4-benzoyl-5-pyrazolone (278.3 mg,1.00 mmol). The mixture was stirred at 333–338 K for 24 h. The solvent was removed under reduced pressure and the residue was recrystallized from ethanol to give the title compound. Yield, 68.9%. m. p. 454–456 K. Anal. Calcd. for C_26_H_24_N_4_O_2_: C, 73.56; H, 5.70; N, 13.20. Found: C,73.63; H,5.81; N,13.07.

Pale-yellow block-like single crystals suitable for X-ray diffraction studies were obtained by slow evaporation from a solution of dichloromethane at room temperature over a period of approximately one month.

### Refinement

H atoms were placed in calculated positions and non-H atoms were refined anisotropically. The remaining H atoms were treated as riding atoms with distances C—H=0.96 Å (CH_3_), 0.93 Å (CH), 0.86 Å (NH), while *U*_iso_(H)=1.20 *U*_eq_(C) for methylidyne and 1.20 *U*_eq_(N) for imino, 1.50 *U*_eq_(C) for methyl. The H atom attached to N atom was located in a different density map and the atomic coordinates allowed to refine freely.

### Crystal data

**Table:**

C_26_H_24_N_4_O_2_	*Z* = 2
*M**_r_* = 424.49	*F*(000) = 448
Triclinic, *P*1	*D*_x_ = 1.286 Mg m^−^^3^
Hall symbol: -P 1	Melting point = 454–456 K
*a* = 7.3550 (6) Å	Mo *K*α radiation, λ = 0.71073 Å
*b* = 11.1609 (13) Å	Cell parameters from 1018 reflections
*c* = 14.9700 (12) Å	θ = 2.9–22.3°
α = 68.536 (1)°	µ = 0.08 mm^−^^1^
β = 76.654 (2)°	*T* = 298 K
γ = 76.182 (2)°	Block-like, yellow
*V* = 1096.46 (18) Å^3^	0.50 × 0.49 × 0.10 mm

### Data collection

**Table:**

Siemens SMART 1000 CCD area-detector diffractometer	3781 independent reflections
Radiation source: fine-focus sealed tube	1861 reflections with *I* > 2σ(*I*)
graphite	*R*_int_ = 0.037
phi and ω scans	θ_max_ = 25.0°, θ_min_ = 2.0°
Absorption correction: multi-scan (*SADABS*; Sheldrick, 1996)	*h* = −8→8
*T*_min_ = 0.960, *T*_max_ = 0.992	*k* = −13→13
5654 measured reflections	*l* = −12→17

### Refinement

**Table:**

Refinement on *F*^2^	Secondary atom site location: difference Fourier map
Least-squares matrix: full	Hydrogen site location: inferred from neighbouring sites
*R*[*F*^2^ > 2σ(*F*^2^)] = 0.077	H-atom parameters constrained
*wR*(*F*^2^) = 0.246	*w* = 1/[σ^2^(*F*_o_^2^) + (0.1078*P*)^2^ + 0.3814*P*] where *P* = (*F*_o_^2^ + 2*F*_c_^2^)/3
*S* = 1.03	(Δ/σ)_max_ < 0.001
3781 reflections	Δρ_max_ = 0.24 e Å^−^^3^
290 parameters	Δρ_min_ = −0.26 e Å^−^^3^
0 restraints	Extinction correction: *SHELXL97* (Sheldrick, 2008), Fc^*^=kFc[1+0.001xFc^2^λ^3^/sin(2θ)]^-1/4^
Primary atom site location: structure-invariant direct methods	Extinction coefficient: 0.014 (5)

### Special details

**Table:**

Geometry. All e.s.d.'s (except the e.s.d. in the dihedral angle between two l.s. planes) are estimated using the full covariance matrix. The cell e.s.d.'s are taken into account individually in the estimation of e.s.d.'s in distances, angles and torsion angles; correlations between e.s.d.'s in cell parameters are only used when they are defined by crystal symmetry. An approximate (isotropic) treatment of cell e.s.d.'s is used for estimating e.s.d.'s involving l.s. planes.
Refinement. Refinement of *F*^2^ against ALL reflections. The weighted *R*-factor *wR* and goodness of fit *S* are based on *F*^2^, conventional *R*-factors *R* are based on *F*, with *F* set to zero for negative *F*^2^. The threshold expression of *F*^2^ > σ(*F*^2^) is used only for calculating *R*-factors(gt) *etc*. and is not relevant to the choice of reflections for refinement. *R*-factors based on *F*^2^ are statistically about twice as large as those based on *F*, and *R*- factors based on ALL data will be even larger.

### Fractional atomic coordinates and isotropic or equivalent isotropic displacement parameters (Å^2^)

**Table:**

	*x*	*y*	*z*	*U*_iso_*/*U*_eq_	
N1	0.5909 (5)	0.6071 (3)	0.1299 (3)	0.0443 (10)	
N2	0.6863 (5)	0.5101 (4)	0.0891 (3)	0.0476 (10)	
N3	0.1377 (5)	0.4355 (4)	0.2977 (3)	0.0498 (11)	
H3	0.1605	0.5047	0.3025	0.060*	
N4	−0.5909 (6)	0.1833 (5)	0.5239 (3)	0.0646 (13)	
O1	0.3193 (4)	0.6395 (3)	0.2404 (2)	0.0505 (9)	
O2	−0.7474 (6)	0.1375 (4)	0.5927 (3)	0.0817 (13)	
C1	0.4246 (6)	0.5767 (4)	0.1894 (3)	0.0403 (11)	
C2	0.4103 (6)	0.4552 (4)	0.1810 (3)	0.0384 (11)	
C3	0.5784 (7)	0.4221 (4)	0.1177 (3)	0.0449 (12)	
C4	0.6428 (7)	0.3052 (5)	0.0868 (4)	0.0602 (15)	
H4A	0.7660	0.3094	0.0477	0.090*	
H4B	0.5548	0.3022	0.0495	0.090*	
H4C	0.6494	0.2281	0.1431	0.090*	
C5	0.6829 (6)	0.7119 (4)	0.1179 (3)	0.0406 (11)	
C6	0.5761 (7)	0.8342 (5)	0.1089 (4)	0.0532 (13)	
H6	0.4470	0.8489	0.1069	0.064*	
C7	0.6606 (8)	0.9334 (5)	0.1030 (4)	0.0600 (15)	
H7	0.5880	1.0155	0.0983	0.072*	
C8	0.8494 (8)	0.9141 (6)	0.1040 (4)	0.0665 (16)	
H8	0.9060	0.9825	0.0998	0.080*	
C9	0.9571 (7)	0.7925 (6)	0.1112 (4)	0.0654 (16)	
H9	1.0869	0.7792	0.1111	0.078*	
C10	0.8737 (7)	0.6911 (5)	0.1184 (4)	0.0533 (14)	
H10	0.9461	0.6089	0.1237	0.064*	
C11	0.2609 (6)	0.3874 (4)	0.2346 (3)	0.0401 (11)	
C12	0.2423 (6)	0.2628 (4)	0.2279 (3)	0.0422 (12)	
C13	0.2792 (7)	0.1492 (5)	0.3045 (4)	0.0532 (13)	
H13	0.3171	0.1517	0.3588	0.064*	
C14	0.2592 (7)	0.0319 (5)	0.2996 (4)	0.0610 (15)	
H14	0.2862	−0.0447	0.3506	0.073*	
C15	0.2010 (7)	0.0263 (5)	0.2221 (4)	0.0613 (15)	
H15	0.1853	−0.0529	0.2202	0.074*	
C16	0.1653 (7)	0.1410 (5)	0.1456 (4)	0.0598 (15)	
H16	0.1251	0.1385	0.0919	0.072*	
C17	0.1881 (6)	0.2558 (5)	0.1483 (4)	0.0500 (13)	
H17	0.1668	0.3313	0.0955	0.060*	
C18	−0.0273 (6)	0.3889 (4)	0.3586 (3)	0.0408 (11)	
C19	−0.0580 (7)	0.3782 (5)	0.4543 (4)	0.0500 (13)	
H19	0.0328	0.3967	0.4792	0.060*	
C20	−0.2214 (6)	0.3404 (4)	0.5147 (4)	0.0488 (13)	
H20	−0.2408	0.3350	0.5796	0.059*	
C21	−0.3560 (6)	0.3105 (4)	0.4794 (3)	0.0426 (12)	
C22	−0.3236 (6)	0.3217 (4)	0.3821 (3)	0.0448 (12)	
H22	−0.4128	0.3012	0.3574	0.054*	
C23	−0.1635 (6)	0.3622 (4)	0.3211 (4)	0.0467 (12)	
H23	−0.1463	0.3717	0.2555	0.056*	
C24	−0.5294 (6)	0.2642 (4)	0.5452 (4)	0.0460 (12)	
C25	−0.6082 (7)	0.3043 (6)	0.6314 (4)	0.0646 (15)	
H25A	−0.7391	0.2944	0.6510	0.097*	
H25B	−0.5983	0.3941	0.6161	0.097*	
H25C	−0.5387	0.2506	0.6833	0.097*	
C26	−0.7905 (10)	0.0306 (7)	0.5759 (6)	0.111 (3)	
H26A	−0.8927	0.0611	0.5388	0.166*	
H26B	−0.8271	−0.0336	0.6370	0.166*	
H26C	−0.6806	−0.0079	0.5404	0.166*	

### Atomic displacement parameters (Å^2^)

**Table:**

	*U*^11^	*U*^22^	*U*^33^	*U*^12^	*U*^13^	*U*^23^
N1	0.051 (2)	0.042 (2)	0.044 (2)	−0.0190 (19)	0.000 (2)	−0.0154 (19)
N2	0.050 (2)	0.053 (2)	0.041 (3)	−0.017 (2)	0.002 (2)	−0.017 (2)
N3	0.051 (2)	0.051 (2)	0.054 (3)	−0.027 (2)	0.005 (2)	−0.021 (2)
N4	0.053 (3)	0.090 (3)	0.056 (3)	−0.038 (2)	0.022 (2)	−0.031 (3)
O1	0.048 (2)	0.055 (2)	0.054 (2)	−0.0193 (16)	0.0039 (17)	−0.0249 (18)
O2	0.076 (3)	0.091 (3)	0.077 (3)	−0.041 (2)	0.017 (2)	−0.028 (2)
C1	0.043 (3)	0.051 (3)	0.032 (3)	−0.019 (2)	−0.005 (2)	−0.014 (2)
C2	0.043 (3)	0.043 (3)	0.031 (3)	−0.017 (2)	−0.002 (2)	−0.011 (2)
C3	0.051 (3)	0.050 (3)	0.039 (3)	−0.018 (2)	−0.005 (2)	−0.015 (2)
C4	0.060 (3)	0.059 (3)	0.067 (4)	−0.020 (3)	0.004 (3)	−0.029 (3)
C5	0.042 (3)	0.044 (3)	0.035 (3)	−0.017 (2)	0.000 (2)	−0.008 (2)
C6	0.046 (3)	0.056 (3)	0.056 (3)	−0.019 (3)	0.001 (3)	−0.015 (3)
C7	0.064 (4)	0.056 (3)	0.061 (4)	−0.024 (3)	0.012 (3)	−0.025 (3)
C8	0.077 (4)	0.074 (4)	0.062 (4)	−0.045 (3)	0.002 (3)	−0.025 (3)
C9	0.047 (3)	0.078 (4)	0.071 (4)	−0.029 (3)	−0.007 (3)	−0.014 (3)
C10	0.045 (3)	0.050 (3)	0.060 (4)	−0.014 (2)	−0.011 (3)	−0.006 (3)
C11	0.048 (3)	0.041 (3)	0.035 (3)	−0.007 (2)	−0.012 (2)	−0.014 (2)
C12	0.043 (3)	0.047 (3)	0.039 (3)	−0.018 (2)	−0.004 (2)	−0.011 (2)
C13	0.057 (3)	0.053 (3)	0.048 (3)	−0.009 (2)	−0.019 (3)	−0.009 (3)
C14	0.072 (4)	0.038 (3)	0.064 (4)	−0.010 (3)	−0.017 (3)	−0.004 (3)
C15	0.064 (3)	0.057 (3)	0.067 (4)	−0.026 (3)	0.010 (3)	−0.028 (3)
C16	0.072 (4)	0.072 (4)	0.053 (4)	−0.036 (3)	0.001 (3)	−0.033 (3)
C17	0.057 (3)	0.057 (3)	0.039 (3)	−0.021 (2)	−0.005 (2)	−0.013 (2)
C18	0.048 (3)	0.037 (2)	0.041 (3)	−0.018 (2)	−0.002 (2)	−0.013 (2)
C19	0.054 (3)	0.062 (3)	0.045 (3)	−0.022 (3)	−0.007 (3)	−0.024 (3)
C20	0.054 (3)	0.058 (3)	0.038 (3)	−0.018 (2)	−0.002 (2)	−0.018 (2)
C21	0.049 (3)	0.036 (2)	0.039 (3)	−0.010 (2)	−0.006 (2)	−0.008 (2)
C22	0.049 (3)	0.051 (3)	0.039 (3)	−0.020 (2)	−0.007 (2)	−0.013 (2)
C23	0.055 (3)	0.056 (3)	0.033 (3)	−0.018 (2)	−0.008 (2)	−0.013 (2)
C24	0.051 (3)	0.045 (3)	0.039 (3)	−0.012 (2)	−0.005 (2)	−0.009 (2)
C25	0.065 (4)	0.077 (4)	0.047 (4)	−0.016 (3)	0.001 (3)	−0.018 (3)
C26	0.113 (6)	0.106 (5)	0.127 (7)	−0.073 (5)	0.034 (5)	−0.052 (5)

### Geometric parameters (Å, °)

**Table:**

N1—C1	1.371 (5)	C12—C17	1.374 (6)
N1—N2	1.401 (5)	C12—C13	1.383 (6)
N1—C5	1.424 (5)	C13—C14	1.381 (7)
N2—C3	1.298 (5)	C13—H13	0.9300
N3—C11	1.321 (6)	C14—C15	1.352 (8)
N3—C18	1.421 (5)	C14—H14	0.9300
N3—H3	0.8600	C15—C16	1.387 (7)
N4—C24	1.263 (6)	C15—H15	0.9300
N4—O2	1.412 (5)	C16—C17	1.348 (6)
O1—C1	1.239 (5)	C16—H16	0.9300
O2—C26	1.423 (7)	C17—H17	0.9300
C1—C2	1.436 (6)	C18—C19	1.361 (6)
C2—C11	1.403 (6)	C18—C23	1.390 (6)
C2—C3	1.435 (6)	C19—C20	1.379 (6)
C3—C4	1.479 (7)	C19—H19	0.9300
C4—H4A	0.9600	C20—C21	1.378 (6)
C4—H4B	0.9600	C20—H20	0.9300
C4—H4C	0.9600	C21—C22	1.383 (6)
C5—C10	1.368 (6)	C21—C24	1.492 (6)
C5—C6	1.379 (6)	C22—C23	1.371 (6)
C6—C7	1.360 (6)	C22—H22	0.9300
C6—H6	0.9300	C23—H23	0.9300
C7—C8	1.356 (7)	C24—C25	1.467 (7)
C7—H7	0.9300	C25—H25A	0.9600
C8—C9	1.378 (8)	C25—H25B	0.9600
C8—H8	0.9300	C25—H25C	0.9600
C9—C10	1.371 (7)	C26—H26A	0.9600
C9—H9	0.9300	C26—H26B	0.9600
C10—H10	0.9300	C26—H26C	0.9600
C11—C12	1.470 (6)		
			
C1—N1—N2	112.5 (3)	C14—C13—C12	119.4 (5)
C1—N1—C5	127.5 (4)	C14—C13—H13	120.3
N2—N1—C5	119.3 (4)	C12—C13—H13	120.3
C3—N2—N1	106.5 (4)	C15—C14—C13	121.3 (5)
C11—N3—C18	129.1 (4)	C15—C14—H14	119.4
C11—N3—H3	115.4	C13—C14—H14	119.4
C18—N3—H3	115.4	C14—C15—C16	118.7 (5)
C24—N4—O2	111.5 (4)	C14—C15—H15	120.6
N4—O2—C26	109.4 (5)	C16—C15—H15	120.6
O1—C1—N1	126.3 (4)	C17—C16—C15	120.8 (5)
O1—C1—C2	129.8 (4)	C17—C16—H16	119.6
N1—C1—C2	103.8 (4)	C15—C16—H16	119.6
C11—C2—C3	131.8 (4)	C16—C17—C12	120.8 (5)
C11—C2—C1	121.9 (4)	C16—C17—H17	119.6
C3—C2—C1	106.1 (4)	C12—C17—H17	119.6
N2—C3—C2	111.0 (4)	C19—C18—C23	119.5 (4)
N2—C3—C4	119.2 (4)	C19—C18—N3	119.2 (4)
C2—C3—C4	129.8 (4)	C23—C18—N3	121.2 (4)
C3—C4—H4A	109.5	C18—C19—C20	121.1 (4)
C3—C4—H4B	109.5	C18—C19—H19	119.5
H4A—C4—H4B	109.5	C20—C19—H19	119.5
C3—C4—H4C	109.5	C21—C20—C19	120.3 (5)
H4A—C4—H4C	109.5	C21—C20—H20	119.9
H4B—C4—H4C	109.5	C19—C20—H20	119.9
C10—C5—C6	120.2 (4)	C20—C21—C22	118.2 (4)
C10—C5—N1	120.6 (4)	C20—C21—C24	120.6 (4)
C6—C5—N1	119.1 (4)	C22—C21—C24	121.1 (4)
C7—C6—C5	119.7 (5)	C23—C22—C21	121.8 (4)
C7—C6—H6	120.2	C23—C22—H22	119.1
C5—C6—H6	120.2	C21—C22—H22	119.1
C8—C7—C6	120.8 (5)	C22—C23—C18	119.1 (4)
C8—C7—H7	119.6	C22—C23—H23	120.4
C6—C7—H7	119.6	C18—C23—H23	120.4
C7—C8—C9	119.6 (5)	N4—C24—C25	124.5 (4)
C7—C8—H8	120.2	N4—C24—C21	114.6 (4)
C9—C8—H8	120.2	C25—C24—C21	120.7 (4)
C10—C9—C8	120.3 (5)	C24—C25—H25A	109.5
C10—C9—H9	119.9	C24—C25—H25B	109.5
C8—C9—H9	119.9	H25A—C25—H25B	109.5
C5—C10—C9	119.4 (5)	C24—C25—H25C	109.5
C5—C10—H10	120.3	H25A—C25—H25C	109.5
C9—C10—H10	120.3	H25B—C25—H25C	109.5
N3—C11—C2	117.6 (4)	O2—C26—H26A	109.5
N3—C11—C12	119.0 (4)	O2—C26—H26B	109.5
C2—C11—C12	123.3 (4)	H26A—C26—H26B	109.5
C17—C12—C13	119.0 (4)	O2—C26—H26C	109.5
C17—C12—C11	122.1 (4)	H26A—C26—H26C	109.5
C13—C12—C11	118.9 (4)	H26B—C26—H26C	109.5
			
C1—N1—N2—C3	3.3 (5)	C3—C2—C11—C12	−5.7 (7)
C5—N1—N2—C3	174.7 (4)	C1—C2—C11—C12	−179.8 (4)
C24—N4—O2—C26	−169.8 (5)	N3—C11—C12—C17	110.5 (5)
N2—N1—C1—O1	174.2 (4)	C2—C11—C12—C17	−73.3 (6)
C5—N1—C1—O1	3.7 (7)	N3—C11—C12—C13	−69.0 (6)
N2—N1—C1—C2	−3.2 (5)	C2—C11—C12—C13	107.2 (5)
C5—N1—C1—C2	−173.8 (4)	C17—C12—C13—C14	−0.6 (7)
O1—C1—C2—C11	0.1 (7)	C11—C12—C13—C14	179.0 (4)
N1—C1—C2—C11	177.4 (4)	C12—C13—C14—C15	−1.2 (8)
O1—C1—C2—C3	−175.3 (5)	C13—C14—C15—C16	1.4 (8)
N1—C1—C2—C3	1.9 (5)	C14—C15—C16—C17	0.0 (8)
N1—N2—C3—C2	−1.8 (5)	C15—C16—C17—C12	−1.8 (8)
N1—N2—C3—C4	−179.4 (4)	C13—C12—C17—C16	2.0 (7)
C11—C2—C3—N2	−174.9 (4)	C11—C12—C17—C16	−177.5 (4)
C1—C2—C3—N2	−0.1 (5)	C11—N3—C18—C19	132.8 (5)
C11—C2—C3—C4	2.3 (8)	C11—N3—C18—C23	−51.4 (7)
C1—C2—C3—C4	177.1 (5)	C23—C18—C19—C20	0.5 (7)
C1—N1—C5—C10	133.8 (5)	N3—C18—C19—C20	176.3 (4)
N2—N1—C5—C10	−36.2 (6)	C18—C19—C20—C21	1.1 (7)
C1—N1—C5—C6	−44.1 (7)	C19—C20—C21—C22	−1.1 (7)
N2—N1—C5—C6	145.9 (4)	C19—C20—C21—C24	177.4 (4)
C10—C5—C6—C7	−1.6 (8)	C20—C21—C22—C23	−0.5 (7)
N1—C5—C6—C7	176.2 (5)	C24—C21—C22—C23	−179.0 (4)
C5—C6—C7—C8	1.3 (8)	C21—C22—C23—C18	2.0 (7)
C6—C7—C8—C9	−0.1 (9)	C19—C18—C23—C22	−2.0 (7)
C7—C8—C9—C10	−0.7 (9)	N3—C18—C23—C22	−177.8 (4)
C6—C5—C10—C9	0.8 (8)	O2—N4—C24—C25	0.9 (7)
N1—C5—C10—C9	−177.0 (5)	O2—N4—C24—C21	176.2 (4)
C8—C9—C10—C5	0.4 (9)	C20—C21—C24—N4	−146.8 (5)
C18—N3—C11—C2	178.2 (4)	C22—C21—C24—N4	31.6 (7)
C18—N3—C11—C12	−5.4 (7)	C20—C21—C24—C25	28.7 (7)
C3—C2—C11—N3	170.6 (5)	C22—C21—C24—C25	−152.9 (5)
C1—C2—C11—N3	−3.6 (6)		

### Hydrogen-bond geometry (Å, °)

**Table:**

*D*—H···*A*	*D*—H	H···*A*	*D*···*A*	*D*—H···*A*
N3—H3···O1	0.86	1.95	2.683 (4)	142
C26—H26C···N4^i^	0.96	2.72	3.643 (9)	163
C9—H9···O1^ii^	0.93	2.67	3.398 (6)	135

Symmetry codes: (i) −*x*−1, −*y*, −*z*+1; (ii) *x*+1, *y*, *z*.
